# Affibody-Derived Drug Conjugates Targeting HER2: Effect of Drug Load on Cytotoxicity and Biodistribution

**DOI:** 10.3390/pharmaceutics13030430

**Published:** 2021-03-23

**Authors:** Haozhong Ding, Tianqi Xu, Jie Zhang, Vladimir Tolmachev, Maryam Oroujeni, Anna Orlova, Torbjörn Gräslund, Anzhelika Vorobyeva

**Affiliations:** 1 Department of Protein Science, KTH Royal Institute of Technology, Roslagstullsbacken 21, 114 17 Stockholm, Sweden; haozhong@kth.se (H.D.); jiezha@kth.se (J.Z.); 2 Department of Immunology, Genetics and Pathology, Uppsala University, Dag Hammarskjölds väg 20, 751 85 Uppsala, Sweden; tianqi.xu@igp.uu.se (T.X.); vladimir.tolmachev@igp.uu.se (V.T.); maryam.oroujeni@igp.uu.se (M.O.); anzhelika.vorobyeva@igp.uu.se (A.V.); 3 Research Centrum for Oncotheranostics, Research School of Chemistry and Applied Biomedical Sciences, Tomsk Polytechnic University, 634050 Tomsk, Russia; anna.orlova@ilk.uu.se; 4 Department of Medicinal Chemistry, Uppsala University, Dag Hammarskjölds väg 14C, 751 23 Uppsala, Sweden

**Keywords:** affibody molecule, human epidermal growth factor receptor 2, HER2, emtansine, DM1, albumin binding domain, DAR, affibody drug conjugate, AffiDC

## Abstract

Affibody molecules hold great promise as carriers of cytotoxic drugs for cancer therapy due to their typically high affinity, easy production, and inherent control of the drug molecules’ loading and spatial arrangement. Here, the impact of increasing the drug load from one to three on the properties of an affibody drug conjugate targeting the human epidermal growth factor receptor 2 (HER2) was investigated. The affibody carrier was recombinantly expressed as a fusion to an albumin-binding domain (ABD) for plasma half-life extension. One or three cysteine amino acids were placed at the C-terminus to which cytotoxic mcDM1 molecules were conjugated. The resulting drug conjugates, Z_HER2_–ABD–mcDM1 and Z_HER2_–ABD–mcDM1_3_, were characterized in vitro, and their biodistribution in mice carrying HER2-overexpressing SKOV3 xenografts was determined. Increasing the drug load from one to three led to a decrease in affinity for HER2, but a significantly more potent cytotoxic effect on SKOV3 cells with high HER2 expression. The difference in cytotoxic effect on other cell lines with high HER2 expression was not significant. In vivo, an increase in drug load led to a 1.45-fold higher amount of cytotoxic mcDM1 delivered to the tumors. The increase in drug load also led to more rapid hepatic clearance, warranting further optimization of the molecular design.

## 1. Introduction

Drug conjugates consisting of a cancer-cell-specific targeting domain coupled to a toxic molecule have been the focus of intense research and clinical development during the last few decades. Antibody–drug conjugates (ADCs), where a monoclonal antibody (mAb) is linked to a cytotoxic drug, have been the most successful for cancer therapy [1]. By combining the specificity of an antibody and the cytotoxic activity of a drug agent, a broad therapeutic window can be obtained by a dramatic reduction in systemic toxicity compared to classical chemotherapy [2]. An example of an ADC is trastuzumab emtansine (T-DM1), consisting of the monoclonal antibody trastuzumab targeting the human epidermal growth factor receptor 2 (HER2) linked with the maytansine derivate DM1 [3]. HER2 is found over-expressed in 20 to 30% of patients with breast cancer, and to a lesser extent in patients with ovarian and gastric cancers [4]. T-DM1 is approved for therapy of HER2-positive metastatic breast cancer by the US Food and Drug Administration. Its mode-of-action relies on binding to HER2 on the cancer cell surface, followed by endocytosis and transport to the lysosomes, where the mAb part is degraded [5]. The cytotoxic drug DM1 subsequently diffuses to the cytosol and acts as an antimitotic agent that binds to tubulin and disrupts microtubule polymerization. This disruption leads to arrest in the G2/M phase of the cell cycle, typically followed by apoptosis [6].

The general difficulties with ADCs include, for example, the high production cost and problems with site-specific drug attachment on the mAb, where the latter leads to a mixture of molecules with variations in drug attachment sites and the drug-to-antibody ratio (DAR). For example, Sun et al. evaluated the pharmacokinetics of T-DM1 with a DAR of up to ten and found that a DAR above six results in accelerated hepatic clearance due to high hydrophobicity [7]. A high drug load has also been correlated to changes in the ADC’s binding properties and physical instability that may affect in vivo efficacy [8]. Additionally, the antitumor potency of ADCs depends on the intracellular concentration of DM1; thus, ADCs with low DAR may suffer from insufficient delivery of the cytotoxic drug to cells with low target-expression [5,9]. The production of ADCs with an optimal DAR is still challenging despite advances in site-specific conjugation chemistries [2]. Besides this, the large size of the mAb-carrier limits tumor penetration, which may result in insufficient delivery to the tumor mass’s interior [10].

As an alternative to mAbs, engineered affinity proteins (EAPs) have been evaluated as carriers of cytotoxic drugs [11]. One class of EAPs is the affibody molecules, which are small (6.5 kDa) scaffold proteins. They are generated by protein engineering techniques, typically resulting in variants with strong affinities and specific binding to selected molecular targets [12]. The small size enables rapid penetration into solid tumor mass, and the typically strong affinity permits good tumor retention [13]. Since naturally occurring cysteine amino acids are lacking in the affibody scaffold [12], cysteines may be inserted at desired positions. They allow for the site-specific conjugation of drug molecules using thiol-directed chemistry, thus achieving a well-controlled DAR and a well-controlled spatial positioning of the drugs. Moreover, the production of affibody molecules can be carried out in relatively simple procaryotic host cells at a high yield [14].

We have previously investigated the properties and therapeutic potential of anti-HER2 affibody molecules equipped with a single mcDM1 drug molecule, so-called AffiDCs [15,16,17]. Given the results on ADCs described above, it is attractive to evaluate the possibility of increasing the drug load for AffiDCs. The impact of an increased number of drugs per affibody targeting moiety has not been explored before to any significant extent. In this study, the protein carrier consisted of an anti-HER2 affibody molecule (Z_HER2:2891_) with a strong affinity to HER2 (equilibrium dissociation constant, K_D_ = 66 pM) [18]. In the tested constructs, Z_HER2:2891_ was fused to an albumin-binding domain (ABD). The ABD is a small protein domain that can form a complex with serum albumin and can be utilized for prolongation of the plasma half-life in vivo [15,16,17,19].

The cytotoxic drug mcDM1 consists of the tubulin polymerization inhibitor DM1 and the non-cleavable maleimidocaproyl (mc) linker. It is relatively hydrophobic, which may drive clearance by the liver. A linker with the amino acid sequence Glu–Glu–Glu has previously been shown to reduce the hydrophobicity and liver uptake of anti-HER2 AffiDCs, when placed next to a C-terminal cysteine where mcDM1 is attached [16]. Therefore, glutamic acids were also added to the constructs in this study with the aim of gaining shielding from the hydrophobic effect of mcDM1 to minimize uptake in the liver. The conjugate with one mcDM1 molecule had a C-terminal ending with the amino acid sequence Glu_3_–Cys, and the conjugate with three mcDM1 drug molecules had the C-terminal end Glu_3_–Cys–Glu_3_–Cys–Glu_3_–Cys. In both cases, mcDM1 molecules were attached to the cysteines. The biophysical properties were investigated, followed by the determination of cytotoxic potential on cell lines with different levels of HER2 expression. The tumor-targeting ability and non-specific uptake in normal organs were investigated in mice carrying HER2 overexpressing ovarian cancer xenografts. This study aimed to examine the effect of drug loading on biochemical characteristics, in vitro cytotoxic efficacy, cellular processing, and in vivo biodistribution by comparing AffiDCs with one or three mcDM1 molecules.

## 2. Materials and Methods

### 2.1. General

The chemicals were purchased from Sigma-Aldrich (St. Louis, MO, USA) or Merck (Darmstadt, Germany), except where otherwise indicated. Restriction enzymes were purchased from New England Biolabs (Ipswitch, MA, USA). Oligonucleotides were synthesized by Integrated DNA Technologies (Leuven, Belgium).

### 2.2. Construction of Genes Encoding Affibody Constructs

The HER2-binding affibody molecule used for targeting was Z_HER2:2891_ [18], herein referred to as Z_HER2_. The gene encoding Z_HER2_–ABD–(Glu_3_Cys)_3_ was PCR-amplified from a plasmid encoding Z_HER2_–ABD–Glu_3_Cys [17] using Phusion polymerase (New England Biolabs). During PCR amplification, NdeI and BamHI restriction enzyme sites were added and a DNA sequence was placed at the N terminus encoding a peptide tag with the sequence His–Glu–His–Glu–His–Glu allowing for radionuclide labeling. The gene was cloned into the pET-21a(+) plasmid vector (Novagen, Madison, WI, USA) using NdeI and BamHI restriction enzymes. Gene integrity was confirmed by DNA sequencing.

### 2.3. Expression and Purification of Affibody Constructs

The affibody/ABD fusion proteins were expressed in *Escherichia coli* BL21 Star (DE3) (New England Biolabs), grown in tryptic soy broth supplemented with 5 g/L yeast extract and 100 μg/mL ampicillin. The cells were grown at 37 °C in shake flasks. Expression was induced by adding 1 mM of isopropyl β-d-1-thiogalactopyranoside (Appolo Scientific, Stockport, UK) when the OD_600_ was between 0.6 and 1.0. The cells were cultured for another 3 h at 37 °C. The cell suspension was centrifuged and the cytoplasmic fraction was released by sonication. The cell lysate was clarified by passage through a 0.45 µm Acrodisc syringe filter (Pall, Port Washington, NY, USA). Human serum albumin-based affinity chromatography on a HiTrap NHS sepharose column (GE Healthcare, Uppsala, Sweden) was performed to isolate the ABD fused affibody constructs. The purification was carried out on an ÄKTA system (GE Healthcare Life Sciences, Uppsala, Sweden), essentially as previously described [20]. Elution was carried out with 500 mM acetic acid (pH = 2.6). The fractions containing affibody fusion proteins were pooled and lyophilized.

### 2.4. Conjugation with mcDM1

The lyophilized affibody fusion proteins were dissolved to a final concentration of 0.1 mM in 100 mM Tris-HCl buffer (pH = 7.85). To prevent the formation of disulfide bonds between the C-terminal cysteines of the constructs, tris (2-carboxyethyl) phosphine (TCEP) was added to a final concentration of 5 mM, followed by incubation for 30 min at 37 °C. Before mcDM1 was added, the protein solution’s pH was adjusted to 6.5 using 1 M HCl. A freshly prepared solution of mcDM1 (Levena Biopharma, San Diego, CA, USA), dissolved in dimethyl sulfoxide (DMSO) to a final concentration of 20 mM, was mixed with the affibody constructs at a molar ratio of 4:3, followed by overnight incubation at room temperature. The conjugation mixture was prepared for reversed-phase high-performance liquid chromatography (RP-HPLC) purification by dilution with 0.1% trifluoroacetic acid in water at 1:1 (*v/v*). The acidified mixture was loaded on a Zorbax C18 SB column (Agilent, Santa Clara, CA, USA) and bound material was eluted by a gradient from 30 to 70% buffer B (0.1% trifluoroacetic acid in acetonitrile) over 40 min at a flow rate of 3 mL/min. The eluted mcDM1-coupled conjugates were lyophilized. 2-iodoacetamide (IAA) was used to cap the three free cysteines in Z_HER2_–ABD–(Glu_3_Cys)_3_ to create the non-toxic control Z_HER2_–ABD–AA_3_. Lyophilized Z_HER2_–ABD–(Glu_3_Cys)_3_ was dissolved in alkylation buffer (0.2 M NH_4_HCO_3_, pH 8.0). TCEP was added to a final concentration of 5 mM, followed by incubation for 30 min at 37 °C. 2-iodoacetamide was added to a final concentration of 10 mM, and the mixture was incubated at room temperature for 30 min in the dark to alkylate the cysteines. The capped protein was purified by RP-HPLC as described above for the affibody–mcDM1 conjugates, followed by lyophilization. The lyophilized protein was dissolved in phosphate-buffered saline (PBS; 10 mM Na-phosphate, 2.7 mM KCl, 137 mM NaCl, pH 7.4) and stored at −80 °C until use.

The concentration of conjugates was determined using the BCA protein assay kit (Thermo Fisher Scientific, Waltham, MA, USA).

Purified conjugates were analyzed by SDS-PAGE (Biorad, Hercules, CA, USA) under reducing conditions. A total of 5 µg was loaded into each lane. The conjugates’ oligomeric state was determined by size-exclusion chromatography by passing the conjugates through a Superdex 75 5/150 column (GE Healthcare, Uppsala, Sweden) at a flow rate of 0.45 mL/min with PBS as a running buffer. The molecular weight of the purified conjugates was measured by electrospray ionization time-of-flight (ESI-TOF) mass spectrometry (Agilent, Santa Clara, CA, USA). Analysis of purity was performed by RP-HPLC (Zorbax 300SB-C18; Agilent) using a gradient from 30 to 60% of 0.1% trifluoroacetic acid in acetonitrile for 30 min, at a flow rate of 1 mL/min.

### 2.5. Affinity Determination

The affinity of the affibody-based conjugates to HER2, human serum albumin (HSA) and mouse serum albumin (MSA) was measured by surface plasmon resonance on a Biacore 3000 instrument (GE Healthcare). All three ligands, HER2-Fc chimera (R & D Systems, Minneapolis, MN, USA), HSA (Novozymes, Bagsvaerd, Denmark) and MSA (Sigma-Aldrich, St. Louis, MO, USA) were coupled to individual flow cells on CM5 chips via amine groups. HBS-EP (10 mM HEPES, 150 mM NaCl, 3 mM EDTA, 0.005% *v/v* surfactant P20, pH 7.4) was prepared as the running buffer and also for the dilution of analytes. The flow rate was 50 µL/min and the temperature was 25 °C. To regenerate the chips, 10 mM HCl was injected for 30 s between each cycle. The binding kinetics were fitted using a 1:1 kinetics model in the Biacore evaluation software.

### 2.6. Cell Culture

AU565, SKBR3, SKOV3, MCF7, BT474, and A549 cell lines were obtained from the American Type Culture Collection (ATCC via LGC Promochem, Borås, Sweden). They were grown in McCoy’s 5A (SKOV3, SKBR3), RPMI-1640 (AU565, BT474), or Dulbecco’s modified Eagle medium (MCF7, A549) (Cytiva Hyclone, Uppsala, Sweden) in a humidified incubator at 37 °C in 5% CO_2_ atmosphere. The media were supplemented with 10% fetal bovine serum (FBS) (20% for BT474) (Sigma-Aldrich) and a mixture of penicillin 100 IU/mL and 100 µg/mL streptomycin.

### 2.7. In Vitro Cytotoxicity Analysis

Serial dilutions of affibody-based conjugates were tested on 5 cell lines, AU565, SKBR3, SKOV3, A549 and MCF7. At 24 h before incubation with conjugates, 5000 cells/well (2000 cells/well for SKOV3) were seeded in a 96-well plate. The cells were then incubated with media containing different concentrations of the conjugates. After 3 days, a colorimetric assay was performed (Cell Counting Kit-8, Sigma-Aldrich) for the determination of cell viability. According to the manufacturer’s protocol, a measurement of A_450_ was carried out to represent cell viability and the acquired absorbance values were plotted against the corresponding conjugate concentrations. The half maximal inhibitory concentration (IC_50_) was determined by Prism (version 9.0.0.) using a log (inhibitor) vs. response-variable slope (four parameters) model (GraphPad Software, La Jolla, CA, USA).

### 2.8. Radiolabeling

Radiolabeling of the conjugates with [^99m^Tc(CO)_3_(H_2_O)_3_]^+^ was performed as described earlier [16]. In brief, technetium-99m was eluted as pertechnetate in 500 μL 0.9% NaCl from a commercial ^99^Mo/^99m^Tc generator. It was incubated in a sealed vial containing CRS kit (PSI, Villigen, Switzerland) at 100 °C for 30 min to generate the [^99m^Tc(CO)_3_(H_2_O)_3_]^+^ (tricarbonyl technetium) precursor. After incubation, 50 μL of the tricarbonyl technetium solution was mixed with 50 μL 0.1 M HCl and 60 μg protein in 50 μL PBS solution. The mixture was incubated at 60 °C for 60 min. The mixture was incubated with a 5000-fold molar excess of histidine at 60 °C for 10 min to remove unbound radioactivity. Subsequently, the mixture was passed through a NAP-5 desalting column (GE Healthcare), pre-equilibrated, and eluted with 1% BSA in PBS to isolate the radiolabeled conjugates. The radiochemical yield and purity were determined by radio-iTLC analysis in PBS and measured using the Cyclone Storage Phosphor System (PerkinElmer, Waltham, MA, USA).

### 2.9. In Vitro Specificity and Cellular Processing

The SKOV3 and BT474 cell lines were seeded in 3 cm Petri dishes (ca. 5 × 10^5^ cells per dish), and a set of three dishes was used for each group. To determine binding specificity, 2 nM of radiolabeled conjugates were incubated with the cells at 37 °C in a 5% CO_2_ atmosphere for 60 min. For the saturation of HER2 receptors on the cells, an additional set of dishes were pre-incubated with 1000 nM of non-radiolabeled conjugates at room temperature for 15 min before incubation with the radiolabeled compound. The medium was discarded, and the cells were washed with PBS and detached by trypsin. The cell suspension was collected, and radioactivity was measured using an automatic gamma spectrometer equipped with a 3 inch NaI (Tl) well detector (1480 Wizard, Wallac, Finland). An unpaired two-tailed t-test was used to analyze the data in Prism (version 9.0.0.).

The radiolabeled conjugates’ internalization rate and cellular processing were studied using a continuous incubation method described earlier [21]. In brief, the radiolabeled conjugates (2 nM) were added to the cells, and the dishes were incubated at 37 °C in a 5% CO_2_ atmosphere. At different time points (1, 2, 4, 6, and 24 h after addition of the conjugates), the medium was collected from one set of dishes, and the cells were washed with PBS. The membrane-bound fraction was collected by incubating the cells in 0.5 mL of glycine buffer containing 4 M urea (pH 2.0) on ice for 5 min. The buffer was collected, and the cells were washed once with the same buffer (0.5 mL) and once with PBS solution (1 mL). The internalized fraction was collected by incubating the cells with 1 M NaOH solution (0.5 mL) at 37 °C for 30 min. The cell lysates were collected and washed once with the same buffer (0.5 mL) and once with PBS solution (1 mL). The radioactivity in each fraction was measured and calculated for the percentage of cell-associated radioactivity. Data were normalized by taking the maximum value of cell-associated radioactivity in each dataset as 100%.

### 2.10. LigandTracer Analysis and Interaction Map Generation

The binding affinity against the HER2-expressing cells of the radiolabeled conjugates was assessed in real-time using a LigandTracer Yellow instrument (Ridgeview Diagnostics, Uppsala, Sweden) as described previously [17]. Increasing concentrations of radiolabeled conjugates (1 nM and 2 nM) were added to the cell culture medium. The binding phase at each concentration was measured for 90 min, followed by over-night incubation with medium only, to measure the dissociation phase. The signal was corrected for nuclide decay, and the binding curves were fitted using TraceDrawer (Ridgeview Instruments, Uppsala, Sweden). Interaction map analysis (Ridgeview Diagnostics, Uppsala, Sweden) was performed to estimate the interaction heterogeneity as described by Altschuh et al. [22].

### 2.11. Biodistribution in Tumor-Bearing Mice

Animal studies were planned in agreement with EU Directive 2010/63/EU for animal experiments and Swedish national legislation concerning laboratory animals’ protection, and were approved by the Ethics Committee for Animal Research in Uppsala, Sweden (animal permission C86/15, approved 28 August 2015).

To study the biodistribution of radiolabeled Z_HER2_–ABD–mcDM1 and Z_HER2_–ABD–mcDM1_3_, 15 female BALB/c nu/nu mice xenografted with SKOV3 cells in the right hind leg were injected with 6 μg of ^99m^Tc-labeled conjugates in 100 μL of 2% BSA in PBS intravenously (i.v.). The injected radioactivity was calculated to give 60 kBq per mouse at the dissection time point. A group of animals (*n* = 3 or 4) was euthanized at 4 and 24 h by intraperitoneal (i.p.) injection of ketamine–xylazine solution (30 μL of solution per gram body weight; ketamine 10 mg/mL; xylazine 1 mg/mL). The organs and tissues were collected, weighed, and measured for radioactivity using an automatic gamma spectrometer. For organs, the percentage of injected dose per gram of sample (%ID/g) was calculated. An unpaired two-tailed t-test was used to analyze the data in Prism (version 9.0.0.).

## 3. Results

### 3.1. Production and Biochemical Characterization of Conjugates

**Figure 1:**
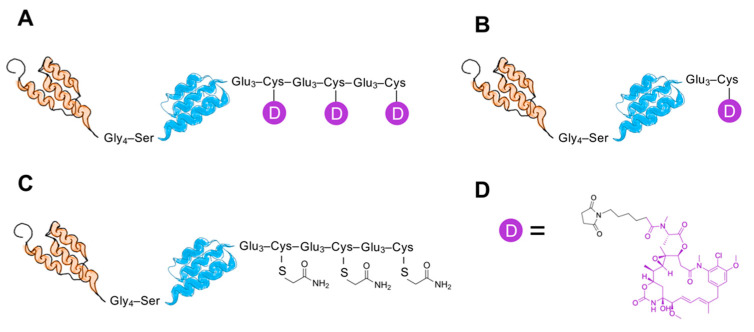
Schematic representation of the conjugates, Z_HER2_–ABD–mcDM1_3_ with drug-to-antibody ratio DAR = 3 (**A**), Z_HER2_–ABD–mcDM1 with DAR = 1 (**B**), and Z_HER2–_ABD–AA_3_ with DAR = 0 (**C**). The representations are not drawn to scale. Z_HER2:2891_, a 58 amino acids human epidermal growth factor receptor 2 (HER2) binding affibody molecule, is represented as a three-helix bundle in orange. The albumin binding domain (ABD), 46 amino acids, is represented as a three-helix bundle in blue. The drug mcDM1 is represented in purple (**D**) with the maleimidocaproyl linker in black.

A HER2-binding affibody-derived drug conjugate (AffiDC) with a DAR of 3, Z_HER2_–ABD–mcDM1_3_, was investigated in this study. Its properties were compared to Z_HER2_–ABD–mcDM1 with a DAR of 1 and the non-toxic control Z_HER2_–ABD–AA_3_. The aim was to understand the impact of drug loading on the characteristics of AffiDCs. The Z_HER2_ and ABD domains were connected with a linker with the amino acid sequence Gly–Gly–Gly–Gly–Ser. The constructs are schematically represented in Figure 1.

**Figure 2:**
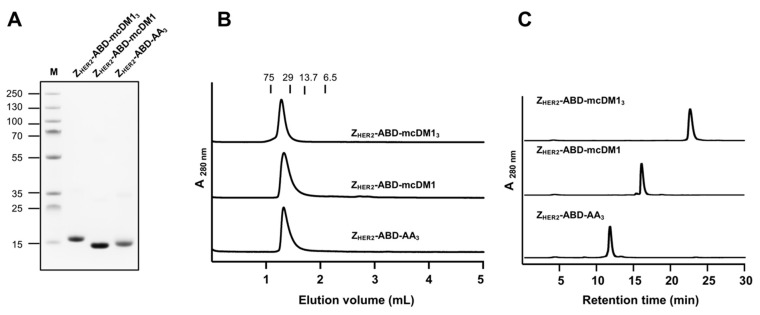
Biochemical characterization of the conjugates. (**A**) Analysis of the anti-HER2 affibody molecules equipped with a single mcDM1 drug molecule and the non-toxic control by sodium dodecyl sulfate polyacrylamide gel electrophoresis (SDS-PAGE) under reducing conditions. The numbers to the left indicate the molecular weights of the marker proteins in lane M (kDa). (**B**) Analysis of the conjugates by size-exclusion chromatography under native conditions. The numbers above the chromatograms indicate the elution volumes of protein standards with different molecular weights (kDa). (**C**) Analysis of the conjugates by reversed-phase high-performance liquid chromatography (RP-HPLC). The conjugates were eluted with a 30 min linear gradient from 30 to 60% acetonitrile in water supplemented with 0.1% trifluoroacetic acid (TFA).

Two fusion proteins, Z_HER2_–ABD–Glu_3_Cys and Z_HER2_–ABD–(Glu_3_Cys)_3_, were recombinantly expressed in a soluble form in *Escherichia coli*. Purification was carried out by a single affinity chromatography step followed by mcDM1 conjugation to the cysteine(s) in the C-terminal end of the proteins, yielding Z_HER2_–ABD–mcDM1 and Z_HER2_–ABD–mcDM1_3_. The non-toxic control was created by alkylating the cysteines in Z_HER2_–ABD–(Glu_3_Cys)_3_, resulting in Z_HER2_–ABD–AA_3_. After a final RP-HPLC purification step, the products were analyzed by SDS-PAGE. As shown in Figure 2A, the conjugates essentially migrated as expected from their molecular weights with no additional bands. The conjugates were analyzed by size-exclusion chromatography under native conditions to investigate the potential formation of multimers (Figure 2B). The conjugates were eluted as single peaks at virtually the expected elution volumes, which shows that they were in a monomeric state and that no multimers were formed. The purity was also determined by analytical RP-HPLC, where the conjugates were analyzed by separation on a C18-column with a linear gradient of acetonitrile in water (Figure 2C). Quantification of the area under curves in the chromatograms showed a purity of >95% for all conjugates. The molecular weights of the conjugates were measured by mass spectrometry. The results showed values exactly matching the expected theoretical molecular weights (Figure S1). From the chromatograms in Figure 2C, it was evident that the non-toxic control Z_HER2_–ABD–AA_3_ (DAR = 0) was eluted before Z_HER2_–ABD–mcDM1 (DAR = 1), which in turn was eluted before Z_HER2_–ABD–mcDM1_3_ (DAR = 3). This result shows the increase in hydrophobicity imparted by mcDM1 on the drug conjugates.

### 3.2. Determination of Binding Affinity to HER2 and Serum Albumins

**Figure 3:**
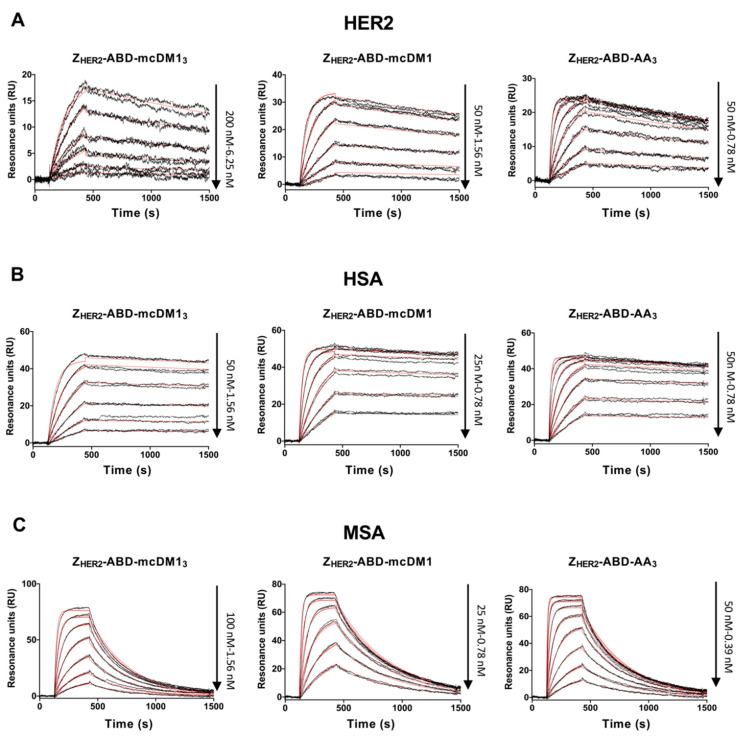
Biosensor analysis. Two-fold serial dilutions of the conjugates indicated over each panel were sequentially injected over flow-cells with immobilized recombinant HER2 (**A**), human serum albumin (HSA) (**B**), and mouse serum albumin (MSA) (**C**). Each concentration was injected twice, and each panel is an overlay of all recorded sensorgrams for each conjugate. The recorded sensorgrams are displayed in black, and the biosensor’s best fitting of the data is shown in red. The concentrations of the injected dilution series are indicated to the right of each panel.

To investigate the affinity to HER2, dilution series of the conjugates were injected into a Biacore biosensor over a surface with immobilized HER2. The kinetic rate constants were derived from the recorded sensorgrams *(*Figure 3A). The three conjugates were found to have comparable dissociation rates (k_d_), ranging from 2.1 × 10^−4^ to 3.0 × 10^−4^ s^−1^. The association rates (k_a_) were different for the three constructs, whereby Z_HER2_–ABD–mcDM1_3_ (DAR = 3) had the slowest association rate (3.4 × 10^4^ 1/Ms), which was ten times lower than the association rate of Z_HER2_–ABD–mcDM1 (DAR = 1), which in turn was three times lower than the association rate of the non-toxic control Z_HER2_–ABD–AA_3_ (DAR = 0). Consequently, the equilibrium dissociation constant (K_D_) was the strongest for Z_HER2_–ABD–AA_3,_ followed by Z_HER2_–ABD–mcDM1 and Z_HER2_–ABD–mcDM1_3_ (Table 1). The cytotoxic drug mcDM1 thus affects the affinity for HER2 by decreasing the association rate.

The interaction of the conjugates with human serum albumin (HSA) and mouse serum albumin (MSA) was similarly investigated in a Biacore biosensor (Figure 3B–C). The kinetic rate constants were derived from the sensorgrams and are displayed in Table 1, together with the calculated K_D_ values. The affinity (K_D_) was stronger to HSA than MSA for the three conjugates due to a slower dissociation rate. Similar to the interaction with HER2, Z_HER2_–ABD–mcDM1_3_ had a weaker affinity than the other two to both HSA and MSA due to a slower association rate.

### 3.3. In Vitro Cytotoxicity Analysis

**Figure 4:**
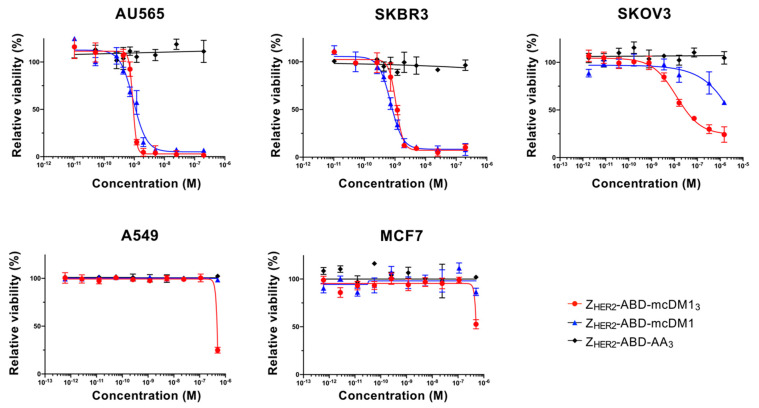
The conjugates’ cytotoxic potential in vitro. The cell lines’ relative viability was determined and plotted against the concentration of the added AffiDCs or the non-toxic control. The half maximal inhibitory concentration (IC_50_) values were determined from the plots when the cells’ relative viability was 50% that of the untreated cells (which was set to 100%). Each concentration was tested in quadruplicate wells, and the mean value is plotted with error bars corresponding to 1 standard deviation (SD).

Various cell lines with a low, medium, or high expression level of HER2 were treated with serial dilutions of the conjugates, followed by a measurement of cell viability to determine their cytotoxic potential (Figure 4, Table 2). For the high-HER2-expressing cell lines, AU565, SKBR3, and SKOV3, both Z_HER2_–ABD–mcDM1 and Z_HER2_–ABD–mcDM1_3_ showed a dose-dependent cytotoxic effect.

The IC_50_ values were similar for the two AffiDC on the AU565 and SKBR3 cells, ranging from 0.7 to 1.1 nM. For SKOV3 cells, Z_HER2_–ABD–mcDM1_3_ was more cytotoxic than Z_HER2_–ABD–mcDM1. The two AffiDCs were considerably less cytotoxic to A549 cells, with medium HER2 expression, and MCF7 cells, with low HER2 expression, than to the highly expressing cell lines. No visible effect on cell viability was detected for any cell lines after treatment with the non-toxic control Z_HER2_–ABD–AA_3_.

### 3.4. Radiolabeling

The conjugates were radiolabeled with ^99m^Tc to allow for the determination of the rate of cellular uptake and for tracking in vivo. The labeling reaction provided a radiochemical yield of over 80% for all conjugates. Purification by desalting/size-exclusion chromatography yielded radiolabeled compounds with >99% radiochemical purity.

### 3.5. In Vitro Specificity and Cellular Processing

**Figure 5:**
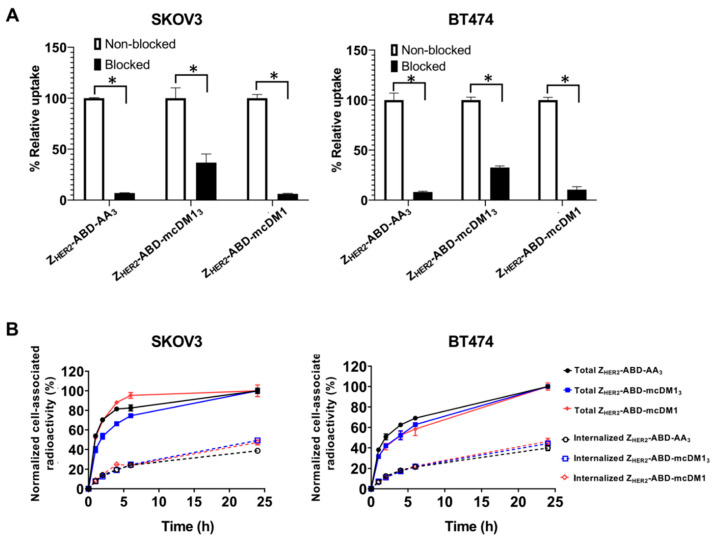
(**A**) In vitro binding specificity of radiolabeled AffiDCs to SKOV3 and BT474 cells, both with high HER2 expression. Non-blocked indicates cells that were incubated with the corresponding radiolabeled compound. Blocked indicates cells pre-incubated with non-radiolabeled conjugate to block available HER2 receptors prior to the incubation with the radiolabeled conjugate. Each bar is the average of three individual measurements, and the error bars in the panels correspond to 1 SD. The star signs (*) correspond to significant differences (*p* < 0.05). (**B**) the cellular processing of radiolabeled AffiDCs by SKOV3 and BT474 cells during continuous incubation over 24 h. Each data point is the average of three individual measurements, and the error bars in the panels correspond to 1 SD.

A blocking experiment was performed to investigate the specificity of the interaction between the radiolabeled conjugates and the HER2-overexpressing cell lines SKOV3 and BT474. The cells were incubated with radiolabeled conjugates with or without pre-incubation with a non-radiolabeled version of the same conjugate. The binding of the radiolabeled conjugates to the cells was significantly decreased (*p* < 0.05) after pre-incubation, where available HER2 receptors were blocked (Figure 5). This result strongly suggested that the HER2 receptor mediated the interaction between the conjugates and the cells.

To determine the uptake and internalization rates, SKOV3 and BT474 cells were incubated with radiolabeled conjugates. The cell-associated radioactivity and internalized fraction were recorded and are shown in Figure 5. For SKOV3 cells, the cellular association of all radiolabeled conjugates was characterized by fast binding, particularly for Z_HER2_–ABD–mcDM1, followed by a plateau phase. The internalization rate was similar for all three conjugates. For BT474 cells, the cellular association was slower and increased during the experiment (24 h) for all three conjugates. The internalization rate was similar for the three conjugates and increased during the whole experiment. The internalized fraction reached 44 ± 4% by 24 h for all conjugates for both cell lines.

### 3.6. Binding of the Conjugates to SKOV3 Cells

**Figure 6:**
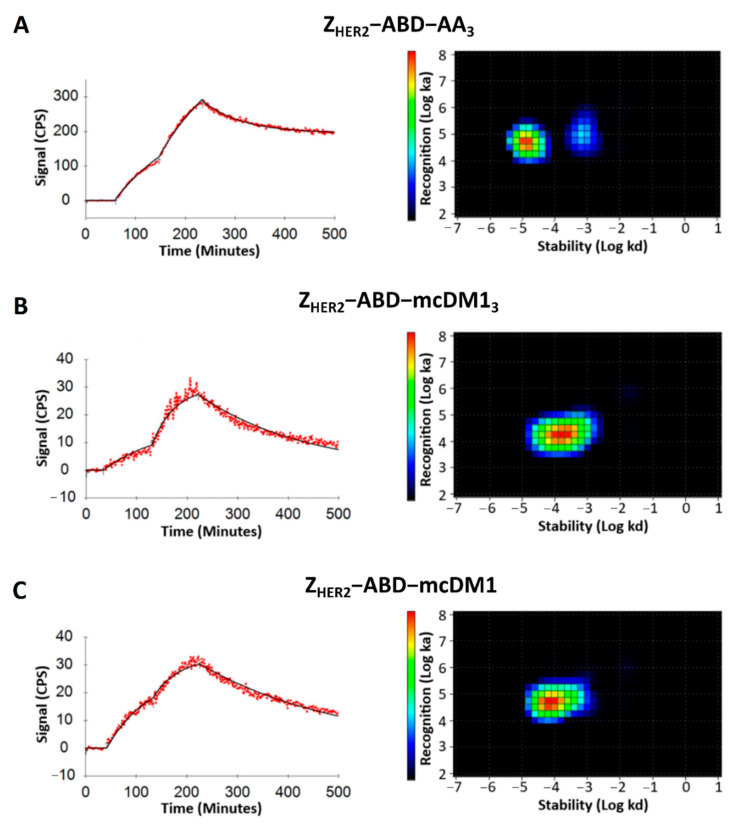
Real-time binding data and interaction map analysis of (**A**) Z_HER2_–ABD–AA_3_, (**B**) Z_HER2_–ABD–mcDM1_3,_ and (**C**) Z_HER2_–ABD–mcDM1 to SKOV3 cells. For the real-time binding analyses to the left in each panel, the cells were incubated with the conjugates at a concentration of 1 nM for 90 min, followed by 2 nM for 90 min to record data for the association phase. At 220 min, the conjugates were removed to record data for the dissociation phase. The interaction maps were derived from the real-time binding analyses and are shown to the right in each panel, with the logarithm of the dissociation rate on the x-axis and the logarithm of the association rate on the y-axis.

The interaction of the radiolabeled conjugates with SKOV3 cells was investigated in real-time using a LigandTracer instrument. The recorded curves fitted well to a one-to-one kinetic model. The real-time interaction data were analyzed with the interaction map method (Figure 6). The homogeneous binding of Z_HER2_–ABD–mcDM1_3_ and Z_HER2_–ABD–mcDM1 to SKOV3 cells was confirmed. For Z_HER2_–ABD–AA_3_, two peaks were seen, representing binding sites with higher affinity (74%) and with lower affinity (26%), respectively. The dissociation equilibrium constant (K_D_) values for conjugates were in the nanomolar range. The impact of drug load on affinity was similar to the impact found in the surface plasmon resonance (SPR) measurements (Figure 3). The highest affinity, 0.4 ± 0.1 nM, was found for Z_HER2_–ABD–AA_3_. The affinity for Z_HER2_–ABD–mcDM1 was 1.76 ± 0.04 nM. The affinity for the triple-loaded Z_HER2_–ABD–mcDM1_3_ was the lowest, 8.1 ± 1.1 nM. Additionally, Z_HER2_–ABD–AA_3_ recognized one more low-affinity binding site on the cells (9.2 ± 3.1 nM).

### 3.7. In Vivo Studies

**Figure 7:**
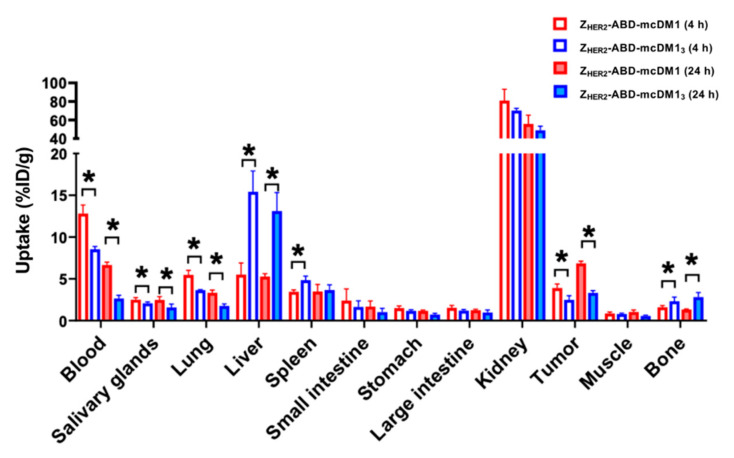
Biodistribution of Z_HER2_–ABD–mcDM1 and Z_HER2_–ABD–mcDM1_3_ in female BALB/c nu/nu mice bearing human SKOV3 xenografts with high HER2 expression. The biodistribution was determined at 4 h (hollow bars) and 24 h (solid bars). The total injected mass was 6 μg/mouse. The data are presented as the mean values (*n* = 3–4) ± 1 SD. The star signs (*) correspond to significant differences (*p* < 0.05).

A biodistribution experiment was performed in mice bearing HER2-expressing SKOV3 xenografts with sampling at 4 h and 24 h p.i. to investigate the behavior of the AffiDCs in vivo, including their specific uptake rate in tumors and non-specific uptake in normal organs (Figure 7). Increasing the DAR from one to three by comparing Z_HER2_–ABD–mcDM1 with Z_HER2_–ABD–mcDM1_3_ was correlated with a lower uptake in the blood at both time points, and thus a more rapid clearance. For both AffiDCs, the tumor uptake increased over time. However, a notable difference was found whereby the tumor uptake of Z_HER2_–ABD–mcDM1_3_ (DAR = 3) was significantly (*p* < 0.05) lower than the uptake of Z_HER2_–ABD–mcDM1 at both time points. The uptake of Z_HER2_–ABD–mcDM1_3_ in liver and bone was also significantly (*p* < 0.05) higher than the uptake of Z_HER2_–ABD–mcDM1 at both time points. The tumor-to-liver (T/L) ratio of Z_HER2_–ABD–mcDM1_3_ was lower compared to the T/L ratio of Z_HER2_–ABD–mcDM1 at both time points (0.2 ± 0.1 vs. 0.7 ± 0.2 at 4 h p.i. and 0.3 ± 0.0 vs. 1.3 ± 0.1% at 24 h p.i., respectively).

## 4. Discussion

This study investigated the impact of drug loading on HER2-specific drug conjugates based on an affibody scaffold protein (AffiDCs). Affibody molecules possess favorable characteristics as carriers of cytotoxic drugs since they typically interact with their intended target in a highly selective manner and with high affinity [12]. A previous study by our group showed that the HER2-specific affibody–drug conjugate Z_HER2_–ABD–mcDM1, carrying one cytotoxic mcDM1 drug molecule, was able to significantly reduce the volume of xenografted SKOV3 tumors in mice, leading to significantly increased survival [17]. However, for antibody–drug conjugates (ADCs), it has been shown that the cytotoxic effect of tubulin polymerization inhibitors, such as DM1, is dependent on the intracellular concentration [5]. From this perspective, a high DAR is desirable. Studies with ADCs have shown that an increase in DAR leads to increased cytotoxic potential but ultimately affects both the clearance rate and affinity for their target negatively [7]. A critical issue is thus to find the optimal DAR [7]. For drug conjugates based on scaffold proteins, the balance between drug loading and in vivo behavior has not been investigated, which motivated us to perform the current study. We hypothesized that increasing the DAR would result in the delivery of a higher dose of the drug to the tumor cells.

A benefit stemming from the cysteine-free affibody backbone is that the DAR and coupling-site specificity of AffiDCs can be precisely regulated by inserting cysteines at desired positions to which the drug molecules can be attached. A previous attempt by Serwotka-Suszczak et al. to load affibody molecules with multiple drug molecules has shown that the separation of partially and fully loaded AffiDCs was difficult [23]. In that study, the tubulin polymerization inhibitor MMAE was conjugated to an HER2-binding affibody carrier, and final purification after conjugation was carried out with hydrophobic interaction chromatography. In contrast, it was straightforward in the present study to isolate the fully loaded AffiDC via an RP-HPLC chromatographic step. A difference between the study by Serwotka-Suszczak et al. and the present study is that non-identical drugs were used, which imparted a different level of hydrophobicity on the AffiDCs, which may have resulted in a more difficult separation process after drug attachment. Differences in the resolution during separation might also have played a role since purification in the HPLC mode in the present study typically results in better resolution in the chromatographic separation step.

A common approach to creating ADCs is to reduce the four interchain disulfide bridges in the antibody, followed by conjugation of the drugs to the reduced cysteines via a thiol–maleimide reaction, analogous to the conjugation reaction used in the present study. This approach can achieve a maximum DAR of 8 in the ADC [24]. However, the purification of the fully loaded antibody from other side products with lower DAR is often carried out by hydrophobic interaction chromatography or size-exclusion chromatography, which usually results in incomplete separation [24,25]. The utilization of a high-resolution RP-HPLC step, as used in the current study, is not available since it would denaturate most antibodies.

The investigation of the interactions between Z_HER2_–ABD–mcDM1_3_ (DAR = 3) and HER2 (Figure 3A), as well as with HER2-overexpressing SKOV3 cells (Figure 6), showed a decreased binding affinity compared to the same interactions involving Z_HER2_–ABD–mcDM1 (DAR = 1) and the non-toxic control Z_HER2_–ABD–AA_3_. In both assays, the on-rate for Z_HER2_–ABD–mcDM1_3_ was slower. This observation could be partially explained by the increase in mass after the conjugation of three mcDM1 molecules, since larger analytes often have a slower on-rate than smaller ones. However, a decrease in target-affinity has been reported in studies on ADCs upon an increase in the drug load [7,24]. In those studies, the percental increase in molecular mass for the ADCs is only minor. Therefore, it is likely that the decrease in affinity is partially a consequence of increasing the drug load of the AffiDCs in this study. The conjugation sites for mcDM1 were placed at the C-terminus of the AffiDC, and it is thus not likely that the mcDM1 is sterically hindering the interaction between the affibody and HER2, since mcDM1 and the affibody are separated by an ABD. Another possible explanation is that the hydrophobic nature of mcDM1 leads to micro-aggregates forming, which would decrease the functional concentration of Z_HER2_–ABD–mcDM1_3_. Such behavior would manifest as a slower association rate in Figure 3A and Figure 6, since the association rate is dependent on the concentration, and an identical dissociation rate, since the dissociation rate is concentration-independent. However, according to Figure 2B, where Z_HER2_–ABD–mcDM1_3_ is analyzed by separation under native conditions, no multimers were formed, and the area under the curve is virtually identical for the three conjugates, thus strongly speaking against the formation of micro-aggregates. At the moment, the mechanism by which multiple mcDM1 drugs influence the interactions involving Z_HER2_ is unclear. It was also observed that the binding affinity of Z_HER2_–ABD–mcDM1_3_ to both serum albumins was decreased five-fold, compared to the other two.

The cytotoxic potency of the DAR = 3 and DAR = 1 AffiDCs was compared on HER2-overexpressing cell lines. On AU565 and SKBR3 cell lines, the IC_50_ values were sub-nanomolar with no significant difference between the high and low DAR variants. For some antibody–drug conjugates, the in vitro potency has been reported to be correlated with the DAR [7]. However, the linker has also sometimes been found to influence the efficacy of ADCs with similar DARs [26]. A reason for the lack of a higher efficiency in Z_HER2_–ABD–mcDM1_3_ could be attributed to the decrease in affinity to HER2. However, a more than 10-fold improved potency of Z_HER2_–ABD–mcDM1_3_ compared to Z_HER2_–ABD–mcDM1 was observed on the SKOV3 cell line, so the difference in potency appears to be cell line-dependent. It is notable that SKOV3 is more resistant to AffiDC poisoning than AU565 and SKBR3, as shown in Figure 4 and an earlier study [17].

The binding specificity test (Figure 5A) demonstrated a significantly higher level of unspecific binding (after HER2 blocking) for Z_HER2_–ABD–mcDM1_3_ compared to other conjugates. This is most likely associated with the higher lipophilicity of Z_HER2_–ABD–mcDM1_3_. A higher lipophilicity typically correlates with a higher unspecific binding, as has been shown, for example, for a series of anti-EGFR affibody molecules conjugated to dyes with different hydrophobicity values [27].

We found that Z_HER2_–ABD–AA_3_ could recognize an additional binding site on SKOV3 cells with lower affinity (Figure 6). The binding of affibody molecules and antibodies to receptors of the HER family receptors with different affinities has been described previously [28]. The formation of altered binding sites was ascribed to the formation of homo- and heterodimers of the receptors on the cell surface, with accompanying conformation changes [28]. The non-toxic control may be capable of recognizing such low-affinity sites, while the mcDM1-conjugated AffiDCs are not.

To achieve a higher in vivo bioavailability of the AffiDCs, an albumin-binding domain (ABD) was included that can bind to serum albumin in blood and prevent kidney filtration. The biodistribution (Figure 7) revealed that the uptake in blood was higher for both AffiDCs than previously reported for AffiDCs lacking the ABD domain [15], thus showing a clear prolongation of the plasma half-life imparted by the ABD. A comparison of the uptake in blood of Z_HER2_–ABD–mcDM1 and Z_HER2_–ABD–mcDM1_3_ at both 4 h and 24 h p.i. showed the more rapid clearance of Z_HER2_–ABD–mcDM1_3_. The difference in kidney uptake between Z_HER2_–ABD–mcDM1 and Z_HER2_–ABD–mcDM1_3_ was not significant. However, there was a significantly higher liver uptake of Z_HER2_–ABD–mcDM1_3_ than Z_HER2_–ABD–mcDM1, suggesting an increased hepatic clearance. In the RP-HPLC analysis (Figure 2C), Z_HER2_–ABD–mcDM1_3_ was eluted later than Z_HER2_–ABD–mcDM1, showing a more hydrophobic character. These observations are consistent with the results derived by Hamblett et al., which showed that an increased DAR was accompanied by an increasing hydrophobicity, which resulted in accelerated plasma clearance [24]. The observations are also consistent with the study by Lyon et al., which showed that rapid clearance was correlated with selective uptake by the liver [29]. Previous studies on affibody molecules and other proteins have shown that hydrophobic patches or positive charges in the proteins promote liver uptake [30]. It is thus likely that mcDM1 promotes liver uptake, and that the incorporation of the glutamic acids near the sites of mcDM1 attachment does not completely shield this effect. In a previous study by our group, a hexa-glutamate spacer next to mcDM1 was analyzed [16]. Even though it did not decrease liver uptake in vivo compared to the same construct with three glutamic acids, it still shortened the elution volume in an RP-HPLC analysis, suggesting a better shielding of the hydrophobic character of mcDM1. It is possible that liver uptake may be alleviated by increasing the number of glutamic acids between the mcDM1 molecules in Z_HER2_–ABD–mcDM1_3_. Another approach might be to attach the mcDM1 molecules further apart on the Z_HER2_–ABD carrier.

Considering the uptake in normal organs, Z_HER2_–ABD–mcDM1_3_ showed a significantly higher (*p* < 0.01) uptake in liver, spleen, and bone in comparison to Z_HER2_–ABD–mcDM1. The receptor-mediated specificity of uptake has previously been tested for ^177^Lu-labeled Z_HER2_–ABD–DOTA [31]. In that study, the conjugate’s design was very similar to the design in this study, with an identical targeting Z_HER2_ moiety and ABD, but with a ^177^Lu-labeled DOTA chelator at the C-terminus instead of mcDM1. For the ^177^Lu-labeled Z_HER2_–ABD–DOTA, the pre-injection of a large excess of non-labeled compound resulted in a significant (more than three-fold) reduction in tumor uptake, but the uptake in normal tissues was not changed. Thus, there was no measurable cross-reactivity of Z_HER2_ with murine HER2. In the present study, the difference in uptake in normal organs therefore resulted from the increase in DAR, and not from the differences in affinity to HER2.

The uptake in tumors increased over time, and at 24 h p.i. the uptake reached 6.8% ID/g for Z_HER2_–ABD–mcDM1 and 3.3% ID/g for Z_HER2_–ABD–mcDM1_3_. Since Z_HER2_–ABD–mcDM1_3_ carried three times as much mcDM1, the delivery of drug molecules to the tumor was 1.45-fold higher for this variant. However, the enhanced delivery of drug molecules to the tumors was compromised by increased uptake in the liver. An important lesson from this study is thus that the development of novel targeted drugs should include the evaluation of their distribution in normal tissues. Such extensive structure–properties relationship studies are necessary to identify molecular design features, which reduce the uptake of high-DAR AffiDC in normal tissues, first and foremost in the liver. The possibility of producing constructs with a well-defined structure and, therefore, with reproducible biodistribution is a favorable feature for AffiDC to have in such studies. The use of radioactive labels, as in this study, facilitates quantitative biodistribution measurements.

## 5. Conclusions

Increasing the DAR of an HER2-binding AffiDC from one to three increases the amount of cytotoxic mcDM1 molecules delivered to implanted tumors by 1.45-fold, but is accompanied by an increase in the non-specific uptake in the liver, bone, and spleen. Further investigations of the molecular design of high-DAR AffiDC are warranted in order to facilitate the highly efficient delivery of drugs to tumors and their low uptake in normal tissues.

## Figures and Tables

**Table 1:** Kinetic parameters and equilibrium dissociation constants.

Analytes	Ligand	k_a_ (M^−1^·s^−1^) ^a^	k_d_ (s^−1^) ^b^	K_D_ (M) ^c^
Z_HER2_–ABD–mcDM1_3_	HER2	3.4 × 10^4^	3.0 × 10^−4^	8.9 × 10^−9^
	HSA	3.0 × 10^5^	6.0 × 10^−5^	2.0 × 10^−10^
	MSA	5.2 × 10^5^	3.0 × 10^−3^	5.8 × 10^−9^
Z_HER2_–ABD–mcDM1	HER2	3.1 × 10^5^	2.1 × 10^−4^	6.9 × 10^−10^
	HSA	1.5 × 10^6^	5.7 × 10^−5^	3.8 × 10^−11^
	MSA	2.4 × 10^6^	2.2 × 10^−3^	9.4 × 10^−10^
Z_HER2_–ABD–AA_3_	HER2	1.0 × 10^6^	3.0 × 10^−4^	3.0 × 10^−10^
	HSA	1.5 × 10^6^	7.7 × 10^−5^	5.2 × 10^−11^
	MSA	2.7 × 10^6^	2.7× 10^−3^	1.0 × 10^−9^

^a^ association rate constant; ^b^ dissociation rate constant; ^c^ equilibrium dissociation constant.

**Table 2:** The conjugates’ cytotoxic potential in vitro.

Cell Lines	IC_50_ (nM)		
	Z_HER2_–ABD–mcDM1_3_	Z_HER2_–ABD–mcDM1	Z_HER2_–ABD–AA_3_
AU565	0.9	0.9	ND ^a^
SKBR3	1.1	0.7	ND
SKOV3	12.4	ND	ND

^a^ Not determined.

## Data Availability

The data generated during the current study are available from the corresponding author upon reasonable request.

## Supplementary Materials

The following are available online at https://www.mdpi.com/1999-4923/13/3/430/s1, Figure S1: Determination of the molecular masses by ESI-TOF mass spectrometry.
