# Supplementary material for: Affibody-Derived Drug Conjugates Targeting HER2: Effect of Drug Load on Cytotoxicity and Biodistribution
Source: Pharmaceutics. 2021 Mar 23;13(3):430. doi: 10.3390/pharmaceutics13030430 (PMC8005000; doi:10.3390/pharmaceutics13030430)
Supplement: Supplementary file 1 [file pharmaceutics-13-00430-s001.pdf]

pharmaceutics

MDPI

Supplementary Materials: Affibody-Derived Drug Conjugates Targeting HER2: Effect of Drug Load on Cytotoxicity and Biodistribution

Haozhong Ding, Tianqi Xu, Jie Zhang, Vladimir Tolmachev, Maryam Oroujeni, Anna Orlova, Torbjörn Gräslund and Anzhelika Vorobyeva

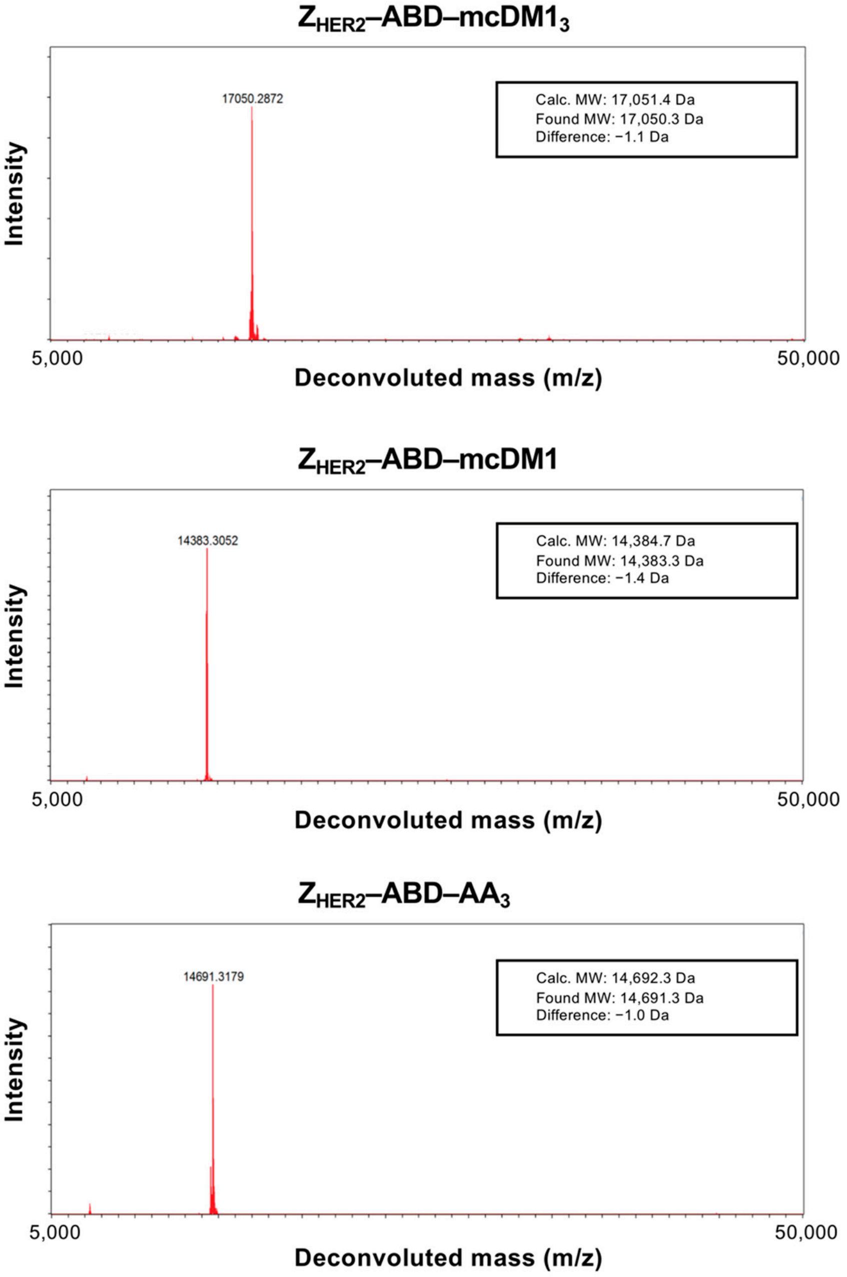

Figure S1. Determination of the molecular masses by ESI-TOF mass spectrometry. The theoretical MW (Calc. MW) and the Found MW of the conjugates are given in the inset, along with the difference between the two.
